# Genetic variation in TLR or NFkappaB pathways and the risk of breast cancer: a case-control study

**DOI:** 10.1186/1471-2407-13-219

**Published:** 2013-05-01

**Authors:** Alexa J Resler, Kathleen E Malone, Lisa G Johnson, Mari Malkki, Effie W Petersdorf, Barbara McKnight, Margaret M Madeleine

**Affiliations:** 1 Program in Epidemiology, Fred Hutchinson Cancer Research Center, 1100 Fairview Avenue North, Seattle, WA 98109, USA; 2 Program in Immunogenetics, Fred Hutchinson Cancer Research Center, 1100 Fairview Avenue North, Seattle, WA 98109, USA; 3 Department of Epidemiology, University of Washington, Health Sciences Building, NE Pacific Street, Seattle, WA 98195, USA; 4 Department of Biostatistics, University of Washington, Health Sciences Building, NE Pacific Street, Seattle, WA 98195, USA; 5 Fred Hutchinson Cancer Research Center, 1100 Fairview Ave N, Mail Stop M4-C308, Seattle, WA 98109, USA

**Keywords:** Breast cancer, Genetic variation, Inflammation, TLR, NFκB

## Abstract

**Background:**

Toll-like receptors (TLRs) and the transcription factor nuclear factor-κB (NFκB) are important in inflammation and cancer.

**Methods:**

We examined the association between breast cancer risk and 233 tagging single nucleotide polymorphisms within 31 candidate genes involved in TLR or NFκB pathways. This population-based study in the Seattle area included 845 invasive breast cancer cases, diagnosed between 1997 and 1999, and 807 controls aged 65–79.

**Results:**

Variant alleles in four genes were associated with breast cancer risk based on gene-level tests: *MAP3K1, MMP9, TANK,* and *TLR9*. These results were similar when the risk of breast cancer was examined within ductal and luminal subtypes. Subsequent exploratory pathway analyses using the GRASS algorithm found no associations for genes in TLR or NFκB pathways. Using publicly available CGEMS GWAS data to validate significant findings (N = 1,145 cases, N = 1,142 controls), rs889312 near *MAP3K1* was confirmed to be associated with breast cancer risk (*P* = 0.04, OR 1.15, 95% CI 1.01–1.30). Further, two SNPs in *TANK* that were significant in our data, rs17705608 (*P* = 0.05) and rs7309 (*P* = 0.04), had similar risk estimates in the CGEMS data (rs17705608 OR 0.83, 95% CI 0.72–0.96; CGEMS OR 0.90, 95% CI 0.80–1.01 and rs7309 OR 0.83, 95% CI 0.73–0.95; CGEMS OR 0.91, 95% CI 0.81–1.02).

**Conclusions:**

Our findings suggest plausible associations between breast cancer risk and genes in TLR or NFκB pathways. Given the few suggestive associations in our data and the compelling biologic rationale for an association between genetic variation in these pathways and breast cancer risk, further studies are warranted that examine these effects.

## Background

Tumor-promoting inflammation has been linked to cancer development in prior research [1-5], and has become recognized as an “enabling characteristic” of other cancer hallmarks such as angiogenesis, cell proliferation and survival, and metastasis [6,7]. The presence of inflammatory messengers in the tumor microenvironment is an important feature of cancer-related inflammation. Many such messengers, including cytokines and chemokines, are produced in response to signaling by transcription factors, such as nuclear factor-κB (NFκB) [1,3,4].

As modulators between inflammation and cancer, NFκB pathway genes play a central role in innate immunity and acute inflammatory response [8,9]. In normal cells, NFκB is activated by various stimuli, such as pathogens and pro-inflammatory cytokines, and controls the expression of multiple target genes, such as *TNF*, *IL6*, and *MMP9*[10-13]. In tumor cells, genetic mutations can compromise NFκB activation, and deregulated expression of genes controlled by NFκB can affect cell proliferation, apoptosis, and cell migration [8,14,15]. Deregulated activation of NFκB has been seen in many common types of cancer, and previous findings suggest that NFκB may be important in breast cancer [16-18].

While NFκB-related pathway genes are critical in innate and adaptive immune responses, genes in toll-like receptor (TLR) signaling pathways are also important as they activate NFκB in addition to other signaling pathways [19]. In normal epithelial cells and cancer cells, TLRs regulate cell proliferation and survival through triggering MAPK and NFκB as well as by mediating the release of cytokines and chemokines [20]. In vitro studies have observed that TLRs are highly expressed in breast cancer cell lines, suggesting that reduced TLR expression could potentially inhibit cell proliferation and survival in breast cancer [21-23].

Further, there is evidence of variants in TLR or NFκB-related pathways affecting gene function. For example, an insertion/deletion (94ins/delATTG) in the promoter of *NFKB1* has been shown to affect transcription [24]. In mice studies, polymorphisms identified in the promoter region, first intron, and 3′ untranslated region (UTR) of *TNF* have been shown to affect production of the cytokine TNF [25]. Likewise, two prior studies found the allele -308A in *TNF* was associated with elevated TNF expression in vitro [25,26]. Additionally, two missense polymorphisms in*TLR4*, rs4986790 (D299G) and rs4986791 (T399I), have been shown to affect the extracellular domain of the TLR4 receptor [27]. Prior studies such as these suggest that polymorphisms in TLR or NFκB-related pathways could affect gene function, and therefore may play a role in cancer susceptibility.

This study examined the association between tagging single nucleotide polymorphisms (tagSNPs) within candidate genes in either TLR or NFκB signaling pathways and breast cancer risk in post-menopausal women. We also conducted an exploratory analysis of multiple genes in TLR or NFκB pathways. We focused this study on older women as circulating levels of pro-inflammatory factors increase with age and breast cancer incidence is highest in this age group.

## Methods

### Study population

Participant recruitment has been described previously [28]. Briefly, cases were women aged 65–79 when diagnosed with invasive breast cancer between April 1997 and May 1999 in the three-county Seattle metropolitan area. Cases were ascertained through the Cancer Surveillance System, a population-based cancer registry included in the Surveillance, Epidemiology and End results (SEER) program [29]. Controls were identified from the general population using Health Care Financing Administration records and were assigned reference dates to match the distribution of diagnosis dates for cases. Controls were frequency matched to cases in 5-year age groups. Of the 1,210 and 1,365 eligible cases and controls, 975 (81%) and 1,007 (74%) completed in-person interviews. DNA was extracted from blood that was collected from 891 cases and 878 controls at the time of interview. Among these participants, adequate DNA was available for 887 cases and 872 controls. Study protocol was approved by the Fred Hutchinson Cancer Research Center institutional review board and written informed consent was obtained from all study participants.

Information that detailed histology, estrogen receptor (ER) status, and progesterone receptor (PR) status was obtained from the Cancer Surveillance System. Tumors were categorized as luminal (ER or PR positive) or non-luminal (ER and PR negative) subtype. Histology was categorized by ICDO codes as ductal (8500), lobular (8520), ductal/lobular (8522), or other (8000, 8481, 8490, 8501, 8512, 8521, 8530, 8980).

### Single nucleotide polymorphism (SNP) selection

As part of a study of breast cancer and inflammation, we examined 1,536 SNPs in pro- or anti-inflammatory genes. For this study, we selected a total of 233 SNPs from 31 genes in TLR or NFκB signaling pathways. The following genes were included: *AZI2*, *IFIH1*, *IKBKE*, *IRAK4*, *IRF3*, *MAP3K1*, *MAP3K7*, *MMP9*, *NFKB1*, *NFKB2*, *RELA*, *RELB*, *TANK*, *TBK1*, *TICAM1*, *TICAM2*, *TIRAP*, *TLR3*, *TLR4*, *TLR7*, *TLR9*, *TNF*, *TNFRSF1A*, *TNFRSF1B*, *TOLLIP*, *TRAF3*, *TRAF6*, *UBE2C*, *UBE3A*, *VISA*, and *ZBP1*. Using the software SNAGGER [30] on publicly available HapMap and SeattleSNPs data, tagSNPs were selected among Caucasians based on an r^2^ value of at least 0.80 and a minor allele frequency (MAF) of 0.05. The tagSNPs were chosen from regions representing the candidate genes plus 4,000 base pairs both 3′ and 5′ of the gene. SNP selection was prioritized based on functional importance, giving SNPs in coding regions priority over those in other regions. To ensure that at least one SNP from each bin would be successfully genotyped, more than one tagSNP was chosen where a bin included more than 10 SNPs. Additionally, coding SNPs within candidate genes with a MAF of at least 0.02 and also SNPs found to be associated with cancer risk in previous studies were included in the panel. For example, rs889312 in the region surrounding *MAP3K1* was selected for analysis based on its significance in prior genome-wide association studies (GWAS) [31,32].

### Genotyping assay

Genotyping was performed on 887 cases and 872 controls using the Illumina GoldenGate multiplex platform (N SNPs = 1,536). Additional assays were run on the KASPAR platform at KBioscience for SNPs not covered on the Illumina platform or that appeared to be failing on Illumina after an interim review (N SNPs = 102). For the current analysis, all 233 SNPs were genotyped on Illumina and four were additionally typed on KASPAR. Of these four SNPs, three failed on Illumina and passed on KASPAR (rs7251, rs10025405, and rs1927907) and one was successfully typed on both platforms (rs5746026) that had a cross-platform concordance of 99.7%. We used results from Illumina to analyze rs5746026 as the call rate was 100%. Replicate aliquots were included for 143 (8%) of the 1,759 participants. Of these replicate-pairs, nine had discordant genotypes of at least 1% among passing SNPs. Monomorphic SNPs or those with call rates less than 90% were excluded from analysis. All SNPs included in this study had Hardy-Weinberg Equilibrium (HWE) p-values greater than 0.001 among Caucasian controls.

### Statistical methods

To account for potential confounding due to population stratification, we used principal components analysis to restrict our sample to 1,652 white women [33]. Briefly, principal components were computed from 872 controls after standardizing the 1,349 SNPs that passed our quality control checks according to the method outlined by Price et al. [33] The first principal component was sufficient to distinguish white from non-white women. Principal components were computed for the entire sample of 1,759 cases and controls after standardizing the 1,349 SNPs to the control population. We determined clusters of white and non-white subjects using the same restriction criteria from the control population. The final study sample consisted of 1,652 individuals that clustered with white women and self-reported their race as white or Hispanic.

Using these 845 cases and 807 controls, the relative risk of breast cancer associated with each SNP was approximated using logistic regression to compute odds ratios (OR) and 95% confidence intervals (CI). All models were adjusted for continuous linear age at reference and were log-additive. However, dominant models were fit when genotype cell counts were less than 5 for either cases or controls. We adjusted for multiple comparisons within a gene by using a minP permutation test with 10,000 replications to assess the significance of each gene [34]. For genes found to be significant (*P* ≤ 0.05) based on the minP permutation test, we used logistic regression to examine the association between SNPs and the risk of ductal histology (N = 565) and luminal breast cancer (N = 744) subtype compared to all controls. These models were adjusted for continuous linear age at reference and were log-additive.

The gene set ridge regression in association studies (GRASS) algorithm was used to conduct exploratory pathway analyses for genes in TLR or NFκB pathways [35]. We examined the association between breast cancer risk and two pathways for genes in our dataset by selecting genes from the Kyoto Encyclopedia of Genes and Genomes (KEGG) “Toll-like receptor signaling pathway” (http://www.genome.jp/kegg/pathway/hsa/hsa04620.html). The first pathway included *TLR3, TLR4, TLR7, TLR9, TIRAP, TICAM1, TICAM2, TOLLIP, IRAK4, TRAF3, TRAF6, MAP3K7, IRF3,* and *IKBKE*. The second pathway included these genes in addition to *NFKB1, NFKB2, RELA,* and *RELB*. Prior to running any models with GRASS, we imputed any missing SNP values. All imputation was performed using BEAGLE 3.3 with a reference panel of phased genotype data from 283 European individuals sequenced by the 1000 Genomes Project [36]. Pathways were determined as significant based on a permutation test with 10,000 replications.

Finally, we used publicly available data from the Cancer Genetics Markers of Susceptibility (CGEMS) Breast Cancer Genome-Wide Association Scan to validate our significant findings [37]. A Holm multiple test procedure was used to compute permutation corrected p-values with 10,000 replications for individual SNPs within significant genes in our data [38]. For SNPs found to be significant (Holm *P* ≤ 0.05), the risk of breast cancer associated with each SNP was computed using logistic regression in the CGEMS data, after adjusting for age in 5-year groups. BEAGLE was used to impute seven SNPs that were not already present within the CGEMS data using phased genotype data from the 1000 Genomes Project as a reference panel. Six SNPs with successful imputation (r^2^ > 0.90) were used for analysis.

All analyses were performed using Stata 11 or R version 2.10.1.

## Results

Cases and controls did not vary substantially in demographic characteristics (Table 1), but there were some key differences for other factors. More cases than controls had a high body mass index (63% vs. 57%, respectively), and family history of breast cancer was more frequent in cases than controls (60% vs. 46%). Specifically, 39% of cases and 29% of controls had a first degree relative with breast cancer. Although a similar fraction of cases and controls had ever had a full-term birth, fewer cases than controls had 3 or more full-term births. Among cases, the majority of tumors were of ductal histology (67%) and luminal subtype (91%).

**Table 1:** Selected characteristics of breast cancer cases and controls

	**Controls (n = 807)**		**Cases (n = 845)**	
	**n**	**%**	**n**	**%**
**Age at reference**				
65–69	253	31.4	264	31.2
70–74	301	37.3	329	38.9
75–79	253	31.4	252	29.8
**Education**				
<HS	108	13.4	103	12.2
HS grad	325	40.3	332	39.3
Some college	232	28.7	275	32.5
College grad+	142	17.6	135	16.0
**Body mass index at reference**				
<18.5	14	1.8	14	1.7
18.5–24.9	322	41.2	293	35.6
25–29.9	244	31.2	290	35.2
30+	202	25.8	226	27.5
**Number of full-term births**				
Nulliparous	73	9.0	76	9.0
1	55	6.8	79	9.3
2	161	20.0	222	26.3
3	221	27.4	216	25.6
4+	297	36.8	252	29.8
**Age at menopause**				
<45	233	28.9	211	25.1
45–49	211	26.1	239	28.4
50–54	241	29.9	260	30.9
55+	122	15.1	131	15.6
**Family history of breast cancer**				
None	236	53.6	190	40.0
1st degree	129	29.3	184	38.7
2nd degree	75	17.0	101	21.3
**Histology**				
Ductal			565	66.9
Lobular			105	12.4
Ductal/lobular			70	8.3
Other			105	12.4
**ER/PR status**				
ER- PR-			73	8.9
ER+ PR-			80	9.8
ER- PR+			7	0.9
ER+ PR+			657	80.4

We examined variation in the risk of breast cancer associated with 233 SNPs representing 31 genes in TLR or NFκB pathways. After correcting for multiple comparisons using the minP permutation test, variation in *MAP3K1, MMP9, TANK,* and *TLR9* was found to be significant at the gene level (Table 2). Results from non-significant genes are presented in Additional file 1: Table S1. The single SNP we assayed in the region surrounding *MAP3K1*, rs889312, was associated with breast cancer risk (OR 1.24, 95% CI 1.06–1.44). In *MMP9* we examined two coding SNPs and one intronic SNP. There was evidence that one of the coding SNPs, rs17576 (Q279R), was associated with an increased risk of breast cancer (OR 1.21, 95% CI 1.04–1.40). Among controls, this SNP was not found to be in high LD with the other two SNPs we examined in *MMP9* (all pairwise r^2^ ≤ 0.50). Of the six SNPs we examined in *TANK*, two were significantly associated with a 20% decreased risk of breast cancer: rs17705608 located in the flanking 5′ UTR and rs7309 located in the 3′ UTR. These SNPs were in moderate LD among controls (r^2^ = 0.67) and had identical relative risk estimates (rs17705608 OR 0.83, 95% CI 0.72–0.96; rs7309 OR 0.83, 95% CI 0.73–0.95). The four other intronic SNPs did not show evidence of affecting breast cancer risk and were not in LD with any other SNPs in this region (all pairwise r^2^ ≤ 0.50). Of the two SNPs we examined in *TLR9*, only the synonymous coding SNP rs352140 (P545P) was associated with breast cancer risk (OR 0.85, 95% CI 0.74–0.97).

**Table 2:** Risk of breast cancer associated with SNPs in TLR or NFκB pathway genes

**Function**		**Maj/Min Allele**	**Controls (n = 807)**			**Cases (n = 845)**			**OR**	**95% CI**		**p**	**SNP perm p**	**Gene wide p**
			**0**	**1**	**2**	**0**	**1**	**2**						
**MAP3K1 (chr 5: 56146657 - 56227736)**														**0.006**
rs889312	Intergenic	A/C	434	318	49	417	337	86	1.24	1.06	1.44	0.006	0.006	
**MMP9 (chr 20: 44070954 - 44078607)**														**0.03**
rs17576	Coding: Q279R	A/G	366	357	78	338	393	106	1.21	1.04	1.40	0.01	0.03	
rs2274756	Coding: R668Q, R668P	G/A	611	178	12	619	204	17	1.14	0.94	1.40	0.19	-	
rs3918262	Intron	A/G	518	246	31	507	282	43	1.18	1.00	1.40	0.05	0.11	
**TANK (chr 2: 161701712 - 161800928)**														**0.04**
rs17705608	Flanking 5′ UTR	A/G	263	404	134	321	406	113	0.83	0.72	0.96	0.01	0.05	
rs7568498	Intron	A/C	558	208	35	569	244	27	1.03	0.86	1.22	0.78	-	
rs1921310	Intron	A/G	449	303	49	504	300	36	0.85	0.72	1.00	0.05	0.17	
rs1267074	Intron	T/A	397	310	94	382	373	84	1.05	0.91	1.22	0.48	-	
rs1267034	Intron	A/G	623	159	19	631	191	18	1.11	0.91	1.35	0.31	-	
rs7309	3′ UTR	A/G	196	392	212	240	421	179	0.83	0.73	0.95	0.008	0.04	
**TLR9 (chr 3: 52230138 - 52235219)**														**0.03**
rs352140	Coding: P545P	A/G	219	391	191	267	406	167	0.85	0.74	0.97	0.02	0.03	
rs187084	Flanking 5′ UTR	A/G	302	362	137	290	381	169	1.13	0.98	1.29	0.08	0.08	

^a^All models are log-additive and adjusted for continuous linear age at reference.

^b^Permutation p-values that are not significant according to the Holm multiple test procedure [38] are not presented.

The results for these four genes were almost identical when analyses were confined to cases with ductal and luminal subtypes respectively (Table 3). For most SNPs, the magnitude of risk associated with each subtype was the same as with the overall risk of breast cancer. Further, only *TLR9* was not significant at the gene level for either ductal or luminal subtypes (minP *P* = 0.14 and 0.09, respectively).

**Table 3:** Risk of ductal and luminal breast cancer associated with SNPs in TLR or NFκB pathway genes

	**Maj/Min Allele**	**Controls (n = 807)**			**Ductal (n = 565)**			**OR**	**95% CI**		**Gene wide*P***	**Luminal (n = 744)**			**OR**	**95% CI**		**Gene wide *P***
		**0**	**1**	**2**	**0**	**1**	**2**					**0**	**1**	**2**				
**MAP3K1**											**0.01**							**0.003**
rs889312	A/C	434	318	49	281	220	60	1.24	1.04	1.46		361	301	77	1.27	1.08	1.48	
**MMP9**											**0.02**							**0.02**
rs17576	A/G	366	357	78	216	276	67	1.24	1.05	1.46		296	340	100	1.23	1.06	1.43	
rs2274756	G/A	611	178	12	424	129	8	1.03	0.82	1.29		541	183	15	1.17	0.95	1.44	
rs3918262	A/G	518	246	31	327	195	35	1.29	1.08	1.56		444	248	40	1.20	1.01	1.43	
**TANK**											**0.002**							**0.004**
rs17705608	A/G	263	404	134	230	256	75	0.78	0.67	0.92		284	358	97	0.82	0.71	0.95	
rs7568498	A/C	558	208	35	385	161	15	0.98	0.80	1.19		495	223	21	1.04	0.87	1.25	
rs1921310	A/G	449	303	49	347	191	23	0.80	0.67	0.96		446	261	32	0.84	0.71	1.00	
rs1267074	T/A	397	310	94	251	253	56	1.07	0.91	1.26		326	336	76	1.09	0.94	1.27	
rs1267034	A/G	623	159	19	412	137	12	1.19	0.95	1.48		546	176	17	1.17	0.96	1.44	
rs7309	A/G	196	392	212	179	264	118	0.78	0.67	0.91		216	375	148	0.80	0.69	0.92	
**TLR9**											**0.14**							**0.09**
rs352140	A/G	219	391	191	180	268	113	0.85	0.73	0.99		234	359	146	0.85	0.74	0.98	
rs187084	A/G	302	362	137	190	254	117	1.16	1.00	1.34		254	337	148	1.13	0.98	1.30	

^a^All models are log-additive and adjusted for continuous linear age at reference.

**Figure 1:**
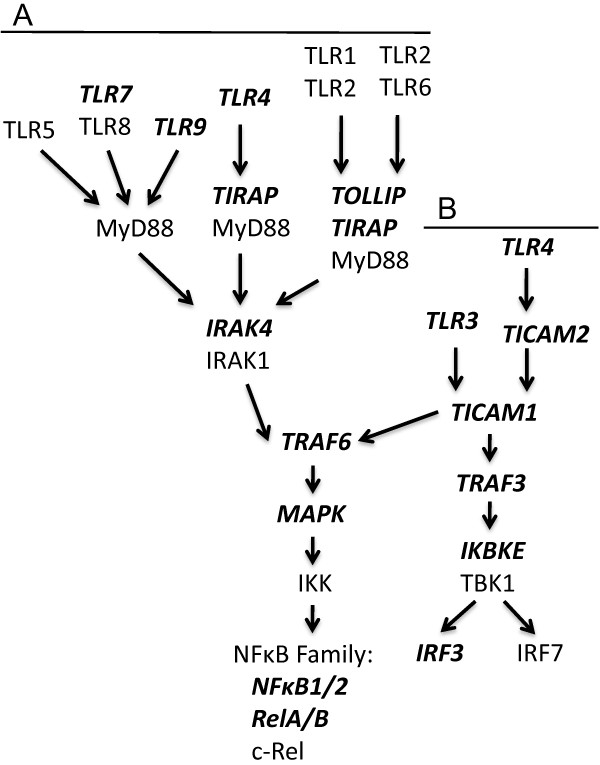
**Toll-like Receptor (TLR) Signaling Pathways.** Classical TLR signaling pathways that result in NFκB activation are either MyD88-dependent (A) or MyD88-independent (B). In MyD88-dependent pathways TLR signaling occurs through the IRAK4/ IRAK1 complex, while in MyD88-independent pathways TLRs signal through TICAM1. TRAF6 then signals to the IKK complex through MAP3K7, which finally leads to NFκB activation. MyD88-independent signaling pathways can also result in the activation of IRF3 or IRF7. Genes assayed by this study are bolded and italicized. This figure was adapted from the KEGG “Toll-like receptor signaling pathway” (http://www.genome.jp/kegg/pathway/hsa/hsa04620.html).

As an exploratory pathway analysis, we used the GRASS algorithm to examine genes in the KEGG “Toll-like receptor signaling pathway” (Figure 1). The first pathway we examined, which included *TLR3, TLR4, TLR7, TLR9, TIRAP, TICAM1, TICAM2, TOLLIP, IRAK4, TRAF3, TRAF6, MAP3K7, IRF3,* and *IKBKE*, was not significant after performing a permutation test (*P* = 0.24). Likewise, after permutation testing the second pathway we examined, which included these same genes in addition to *NFKB1, NFKB2, RELA,* and *RELB,* was not significant (*P* = 0.28)*.*

We attempted to validate significant findings by assessing the risk of breast cancer associated with SNPs from our Seattle study using data from the CGEMS GWAS repository. Most SNPs found to be significant in our data were not found to be significant in the CGEMS data (Table 4). Only rs889312 from the region near *MAP3K1* was replicated, and without correction for multiple comparisons (*P* = 0.04 in CGEMS), with the suggestion of a slight increased risk of breast cancer (OR 1.15, 95% CI 1.01–1.30). Although the associations with breast cancer were of similar magnitude and direction for most SNPs when comparing the two datasets, the risk of breast cancer associated with rs352140 in *TLR9* was in the opposite direction (OR 1.06, 95% CI 0.94–1.19) from that found in our data (OR 0.85, 95% CI 0.74–0.97).

**Table 4:** Risk of breast cancer associated with SNPs in the CGEMS GWAS data

	**Maj/Min Allele**	**Controls (n = 1142)**			**Cases (n = 1145)**			**OR**^**a**^	**95% CI**		***P***
		**0**	**1**	**2**	**0**	**1**	**2**				
**MAP3K1**											
rs889312	A/C	607	447	88	552	499	94	1.15	1.01	1.30	0.04
**MMP9**											
rs17576	A/G	464	542	136	452	541	152	1.05	0.93	1.19	0.39
**TANK**											
rs17705608	A/G	412	535	195	442	538	165	0.90	0.80	1.01	0.07
rs7309	G/A	303	541	286	312	570	241	0.91	0.81	1.02	0.10
**TLR9**											
rs352140	T/C	356	560	226	337	572	236	1.06	0.94	1.19	0.36

^a^All models are log-additive and adjusted for age in 5 year groups.

## Discussion

We found that the risk of breast cancer was associated with genetic variation in four genes in either TLR or NFκB pathways: *MAP3K1, MMP9, TANK,* and *TLR9*. Results were unchanged within cases with ductal or luminal subtypes. However, after replicating our results using the CGEMS GWAS data, only rs889312 from the region near *MAP3K1* was associated with breast cancer risk.

*MAP3K1* is a key player in TLR signaling pathways and produces downstream signaling for the NFκB pathway as well as the ERK and JNK kinase pathways [39,40]. Our finding for rs889312 is consistent with previous results, as variants near *MAP3K1* have been found to be significant in three prior GWAS studies [31,32,41]. Easton et al. found rs889312 to be significantly associated with breast cancer risk in 4,398 breast cancer cases and 4,316 controls [31]. They confirmed this finding in 21,860 cases and 22,578 controls using data from the Breast Cancer Association Consortium (BCAC) GWAS, which combined 22 case-control studies. Further, the magnitude of risk in the Easton et al. study was comparable to that found in our study population for rs889312 (OR 1.13, 95% CI 1.10–1.16). In a more recent GWAS, Turnbull et al. also found that rs889312 was associated with an increased risk of breast cancer among 12,576 cases and 12,223 controls (OR 1.22, 95% CI 1.14–1.30) [32]. In the CGEMS GWAS, they did not directly assess rs889312 but they found that rs16886165 significantly affected the risk of breast cancer after combining 5,440 cases and 5,283 controls [41]. After we imputed rs889312 in the CGEMS data, we found it was in moderate LD with rs16886165 (r^2^ = 0.68). A candidate gene study, which used 1,267 Dutch breast cancer cases and 20,973 controls from the BCAC GWAS, did not find rs889312 to significantly affect breast cancer risk (OR 1.03, *P* = 0.72), though they did find that this SNP was associated with lymph-node status (*P* = 0.04) [42]. However, as the population used in this Dutch study was a subset of the BCAC GWAS, it is important to note that their results correlate with those from the BCAC GWAS.

We also investigated variation in *MMP9*, as MMPs influence cancer progression and contribute to tumor angiogenesis, growth, and metastasis by degrading the extracellular matrix and activating growth factors [43]. *MMP9* expression is regulated by NFκB [44], and in one study was shown to be correlated with NFκB activation in patients with squamous cell carcinoma of the uterine cervix [45]. Although no GWAS studies have found SNPs in *MMP9* to affect breast cancer risk, many prior studies have published results that support an association between *MMP9* and breast cancer risk. Two previous analyses of expression found *MMP9* plasma concentrations were greater in breast cancer cases compared to controls [46,47]. In a Polish study of 270 breast cancer cases and 300 controls, Przybylowska et al. found increased levels of *MMP9* in tumor samples compared to normal breast tissue and an increased risk of breast cancer associated with the T allele for rs3918242 in *MMP9* (OR 2.6, 95% CI 1.3–4.9) [48]. In a candidate gene study of 959 cases and 952 controls from Sweden, Lei et al. found a non-significant increased risk of breast cancer associated with TT homozygotes for rs3918242 (OR 1.88, 95% CI 0.97–3.63) [49]. However, findings from two prior meta-analyses of case-control studies (one which used 15,328 cases and 15,253 controls) showed no association between rs3918242 and breast cancer risk [50,51]. Our study is the only one to date to find an association between the coding SNP rs17576 in *MMP9* and breast cancer risk.

Another NFκB gene we investigated was *TANK* (also known as *TRAF2*), which is a critical upstream component in the NFκB activation pathway and therefore could be a factor that relates to inflammation as well as cancer development and progression [12,13,52,53]. Although two SNPs in *TANK* (rs17705608 and rs7309) were significantly associated with breast cancer risk in our study sample, interestingly no prior GWAS or candidate gene studies have reported on genetic variants in *TANK* affecting the risk of breast cancer. In the CGEMS GWAS data, neither of these SNPs was strongly associated with breast cancer risk (rs17705608 OR 0.90, 95% CI 0.80–1.01; rs7309 OR 0.91, 95% CI 0.81–1.02).

As TLR pathways are central in tissue repair and regeneration [19,54,55], we investigated several TLRs including *TLR9*. No GWAS studies to date have found that breast cancer risk is influenced by variants in *TLR9*. We found that rs352140 in *TLR9* was associated with breast cancer risk (OR 0.85, 95% CI 0.74–0.97). Although this SNP is synonymous and does not alter the protein sequence, it could affect the protein via perturbations in mRNA splicing and stability, altered structure of mRNA, and (though less well-established) effects on protein folding [56]. Our result for rs352140 was in contrast to a small Croatian study that found no association in 130 breast cancer cases and 101 controls (and which may have been underpowered to detect this association) [57]. However, expression studies have found breast cancer patients to have high levels of *TLR9*[21,58,59]. Berger et al found that women with breast cancer had higher circulating levels of *TLR9* compared to controls, and that *TLR9* mRNA expression was correlated with NFκB activity in breast cancer patients [58]. Therefore, future studies should continue to assess the relationship between polymorphisms in *TLR9* and breast cancer risk.

In exploratory pathway analyses we did not observe an association between TLR-NFκB related genes and breast cancer risk. Although the results from these exploratory pathway analyses do not suggest that breast cancer risk is affected by combined variation in the genes that we examined from the KEGG “Toll-like receptor signaling pathway”, this study may have been limited to detect such an association given our sample size and the absence of some key genes within this pathway (such as *MyD88*, *TLR1*, and *TLR2*). Given the biologic plausibility that genes within this pathway could affect cancer development and progression, it would be of interest for further studies to include pathway analyses, particularly those that have larger sample sizes, improved coverage of SNP variation, and other sources of variation such as epigenetic influences.

Although this study suggested variation in four genes*, MAP3K1, MMP9, TANK,* and *TLR9*, may affect the risk of breast cancer, previous studies have observed associations for other genes in TLR or NFκB pathways. For example, prior studies have identified polymorphisms in *TLR4* (rs4986790) [60] and *TNF* (rs361525 and rs1800629) [61-63] that affect breast cancer risk. A prior study, that included a subset of the participants in this study, found breast cancer risk was associated with a UTR 5′ flanking SNP (rs2009658) in lymphotoxin alpha (*LTA*) (OR 1.2, 95% CI 1.1–1.4) as well as a nonsynonomous coding SNP (rs767455) in the TNF receptor *TNFRSF1A* (OR 1.2, 95% CI 1.1–1.4) [64].

There were some limitations to this study that should be considered in the interpretation of our results. Our sample size may not have been sufficient to capture the true level of association between genetic variants with low frequency and breast cancer risk. Also, the assays we used may have misclassified or failed to detect variation in the genes we analyzed. However, misclassification is not likely a problem as the repeat samples were highly concordant. There could also be missed variation due to incomplete coverage of genes or due to our limited number of SNPs. It is also possible that we did not characterize important variation in these genes, since particular variants, such as deletions, variants in repeat regions, and copy number variants, were not detectable on the platforms we used for genotyping. Another limitation is that we did not genotype variants for every gene in TLR or NFκB related pathways. Therefore, potentially important associations between key genes in these pathways may have been missed. In addition, although we attempted to control for potential population stratification by restricting our sample to white women using principal components analysis, it is possible our analyses were subject to uncontrolled confounding from admixture.

There were a number of strengths to this study. For one, our well-characterized study population is representative of post-menopausal women at risk of breast cancer in the Seattle metropolitan area. Also the population-based controls are representative of those at risk of disease. Further, our study sample is consistent with other populations that have been used to analyze breast cancer risk, raising the likelihood that associations from this study are generalizable to similar populations. Another strength of this study was our use of a tagSNP approach that maximized genetic coverage. Finally, by using data from the CGEMS GWAS to validate our findings we were able to draw stronger conclusions regarding the association between genetic variants in TLR or NFκB pathways and breast cancer risk.

## Conclusions

Overall, the results of this study do not suggest a strong association between breast cancer risk and the SNPs in the candidate genes we analyzed in TLR or NFκB pathways. Despite our findings, there is a compelling biologic rationale for an association between genetic variation in these pathways and breast cancer risk. Given the few suggestive associations in our data and results from prior studies that implicate plausible associations between breast cancer risk and genes in TLR or NFκB pathways, further studies are warranted that examine these effects.

## Competing interests

The authors declare that they have no competing interests.

## Authors’ contributions

MMM and KEM provided the concept of the study as well as funding. MM and EWP provided laboratory methodology and expertise in evaluating the assay results. The analysis plan was developed by KEM, LGJ, AJR, and MMM under the direction of BM. Data analysis was performed by AJR and LGJ. AJR conducted the literature review and prepared the manuscript, including the Background, Materials and Methods, Results, Discussion, and Conclusions sections. All authors contributed substantially to revisions toward the final manuscript. All authors read and approved the final manuscript.

## Pre-publication history

The pre-publication history for this paper can be accessed here:

http://www.biomedcentral.com/1471-2407/13/219/prepub

## Supplementary Material

Additional file 1: Table S1 Risk of Breast Cancer Associated with SNPs in Non-significant TLR or NFκB Pathway Genes. Click here for file
