# Supplementary material for: Genetic variation in TLR or NFkappaB pathways and the risk of breast cancer: a case-control study
Source: BMC Cancer. 2013 May 1;13:219. doi: 10.1186/1471-2407-13-219 (PMC3651307; doi:10.1186/1471-2407-13-219)
Supplement: Additional file 1: Table S1 — Risk of Breast Cancer Associated with SNPs in Non-significant TLR or NFκB Pathway Genes. [file 1471-2407-13-219-S1.doc]

**Supplementary Table 1. Risk of Breast Cancer Associated with SNPs in Non-significant TLR or NFκB Pathway Genes**

|  |  |  |  |  | Controls | | |  | Cases | | |  |  |  |  |
| --- | --- | --- | --- | --- | --- | --- | --- | --- | --- | --- | --- | --- | --- | --- | --- |
|  |  |  |  |  | (n = 807) | | |  | (n = 845) | | |  |  |  |  |
|  |  |  | Function | Maj / Min Allele | 0 | 1 | 2 |  | 0 | 1 | 2 | OR | 95% CI | | gene wide *P* |
| **AZI2 (chr 3: 28339090-28365579)** | | | |  |  |  |  |  |  |  |  |  |  |  | **0.62** |
|  | rs7613767 |  | flanking 3' UTR | G / A | 408 | 344 | 47 |  | 438 | 345 | 56 | 0.99 | 0.85 | 1.16 |  |
|  | rs4393865 |  | intron | G / A | 439 | 304 | 58 |  | 479 | 309 | 51 | 0.91 | 0.78 | 1.07 |  |
|  | rs12054402 | * | intron | A / G | 696 | 103 | 2 |  | 734 | 101 | 5 | 0.96 | 0.72 | 1.28 |  |
|  | rs7649498 |  | flanking 5' UTR | C / A | 239 | 419 | 143 |  | 256 | 436 | 148 | 0.98 | 0.85 | 1.13 |  |
| **IFIH1 (chr 2: 162831835-162883285)** | | | |  |  |  |  |  |  |  |  |  |  |  | **0.69** |
|  | rs1990760 |  | coding (A946T) | A / G | 310 | 365 | 125 |  | 315 | 388 | 136 | 1.04 | 0.90 | 1.19 |  |
|  | rs3747517 |  | coding (H843R) | G / A | 427 | 321 | 53 |  | 444 | 325 | 69 | 1.05 | 0.90 | 1.22 |  |
|  | rs10930046 |  | coding (H460R) | A / G | 780 | 21 | 0 |  | 821 | 17 | 0 | 0.76 | 0.40 | 1.46 |  |
|  | rs17715343 |  | intron | C / G | 667 | 125 | 9 |  | 712 | 123 | 5 | 0.88 | 0.69 | 1.12 |  |
| **IKBKE (chr 1: 204710419-204736845)** | | | |  |  |  |  |  |  |  |  |  |  |  | **0.12** |
|  | rs6657352 | * | flanking 5' UTR | G / A | 701 | 97 | 2 |  | 724 | 110 | 4 | 1.12 | 0.84 | 1.49 |  |
|  | rs1930437 |  | intron | C / A | 217 | 399 | 185 |  | 223 | 421 | 195 | 1.01 | 0.88 | 1.16 |  |
|  | rs34253940 |  | intron | A / C | 308 | 375 | 118 |  | 331 | 399 | 110 | 0.95 | 0.82 | 1.09 |  |
|  | rs1539243 |  | coding (I67I) | G / A | 566 | 219 | 16 |  | 577 | 240 | 23 | 1.11 | 0.92 | 1.33 |  |
|  | rs2297546 |  | intron | C / G | 323 | 363 | 115 |  | 286 | 414 | 140 | 1.20 | 1.04 | 1.38 |  |
|  | rs1953090 |  | intron | A / C | 431 | 316 | 54 |  | 465 | 323 | 52 | 0.95 | 0.81 | 1.11 |  |
|  | rs11117909 |  | intron | G / A | 622 | 167 | 11 |  | 626 | 203 | 11 | 1.16 | 0.94 | 1.43 |  |
|  | rs2297545 |  | coding (T239T) | G / A | 482 | 279 | 39 |  | 493 | 295 | 50 | 1.08 | 0.91 | 1.27 |  |
|  | rs12724769 |  | intron | A / G | 192 | 411 | 197 |  | 236 | 403 | 196 | 0.90 | 0.78 | 1.03 |  |
|  | rs41299005 |  | intron | G / A | 643 | 147 | 9 |  | 714 | 121 | 5 | 0.73 | 0.58 | 0.93 |  |
|  | rs11118087 |  | intron | G / A | 421 | 315 | 65 |  | 436 | 331 | 73 | 1.03 | 0.89 | 1.20 |  |
|  | rs11118092 |  | intron | G / A | 317 | 379 | 105 |  | 354 | 366 | 120 | 0.97 | 0.84 | 1.12 |  |
|  | rs11118132 |  | intron | G / C | 349 | 344 | 108 |  | 324 | 396 | 120 | 1.13 | 0.98 | 1.30 |  |
|  | rs12728136 |  | intron | G / A | 588 | 198 | 15 |  | 628 | 193 | 19 | 0.96 | 0.79 | 1.17 |  |
|  | rs41299852 | * | intron | A / G | 764 | 37 | 0 |  | 801 | 38 | 1 | 1.01 | 0.63 | 1.59 |  |
|  | rs17434047 | * | 3' UTR | G / A | 751 | 50 | 0 |  | 779 | 60 | 1 | 1.18 | 0.80 | 1.73 |  |
|  | rs2336940 |  | intron | G / C | 572 | 212 | 17 |  | 613 | 199 | 28 | 0.99 | 0.82 | 1.19 |  |
|  | rs3748022 |  | coding (P713L) | G / A | 500 | 270 | 31 |  | 503 | 279 | 58 | 1.17 | 1.00 | 1.38 |  |
|  | rs15672 |  | UTR | G / A | 207 | 398 | 196 |  | 255 | 418 | 167 | 0.83 | 0.72 | 0.95 |  |
|  | rs11576885 |  | flanking 3' UTR | G / C | 323 | 368 | 110 |  | 362 | 369 | 108 | 0.92 | 0.80 | 1.06 |  |
| **IRAK4 (chr 12: 42439047-42468166)** | | | |  |  |  |  |  |  |  |  |  |  |  | **0.23** |
|  | rs4251460 |  | intron | A / C | 646 | 137 | 17 |  | 691 | 135 | 14 | 0.91 | 0.73 | 1.13 |  |
|  | rs1461567 |  | intron | G / A | 409 | 330 | 62 |  | 452 | 320 | 67 | 0.94 | 0.80 | 1.09 |  |
|  | rs4251520 |  | intron | T / C | 570 | 163 | 11 |  | 618 | 136 | 10 | 0.81 | 0.64 | 1.01 |  |
|  | rs4251545 |  | coding (A428T) | G / A | 665 | 121 | 15 |  | 711 | 121 | 8 | 0.86 | 0.68 | 1.09 |  |
|  | rs1141168 |  | flanking 3' UTR | A / G | 195 | 371 | 175 |  | 230 | 372 | 156 | 0.87 | 0.75 | 1.00 |  |
|  | rs4251565 | * | flanking 3' UTR | A / G | 688 | 109 | 4 |  | 741 | 94 | 5 | 0.82 | 0.61 | 1.09 |  |
| **IRF3 (chr 19: 54854641-54860926)** | | | |  |  |  |  |  |  |  |  |  |  |  | **0.31** |
|  | rs7251 |  | coding (S427T) | G / C | 345 | 322 | 80 |  | 370 | 327 | 71 | 0.92 | 0.79 | 1.07 |  |
|  | rs2304207 |  | intron | C / G | 562 | 212 | 27 |  | 603 | 224 | 13 | 0.88 | 0.72 | 1.06 |  |
|  | rs2304204 |  | UTR | A / G | 454 | 294 | 53 |  | 487 | 311 | 42 | 0.92 | 0.78 | 1.08 |  |
| **MAP3K7 (chr 6: 91282074-91353628)** | | | |  |  |  |  |  |  |  |  |  |  |  | **0.99** |
|  | rs157693 |  | flanking 3' UTR | C / G | 279 | 400 | 120 |  | 303 | 401 | 136 | 1.00 | 0.87 | 1.15 |  |
|  | rs806286 |  | flanking 3' UTR | A / G | 416 | 318 | 67 |  | 436 | 331 | 73 | 1.01 | 0.87 | 1.18 |  |
|  | rs1231646 |  | flanking 3' UTR | G / A | 387 | 346 | 68 |  | 409 | 361 | 70 | 0.99 | 0.85 | 1.15 |  |
|  | rs2172710 | * | 3' UTR | G / A | 758 | 42 | 1 |  | 791 | 48 | 1 | 1.09 | 0.72 | 1.66 |  |
|  | rs806284 |  | intron | C / A | 417 | 317 | 67 |  | 438 | 333 | 69 | 1.00 | 0.86 | 1.16 |  |
|  | rs13208824 |  | intron | C / A | 604 | 183 | 14 |  | 636 | 189 | 15 | 0.99 | 0.81 | 1.21 |  |
| **NFKB1 (chr 4: 103641518 - 103757507)** | | | |  |  |  |  |  |  |  |  |  |  |  | **0.33** |
|  | rs3774932 |  | intron | G / A | 259 | 393 | 149 |  | 262 | 406 | 172 | 1.06 | 0.93 | 1.22 |  |
|  | rs3774933 |  | intron | A / G | 284 | 382 | 135 |  | 306 | 403 | 128 | 0.95 | 0.82 | 1.09 |  |
|  | rs3774934 |  | intron | G / A | 650 | 144 | 7 |  | 677 | 157 | 6 | 1.02 | 0.81 | 1.29 |  |
|  | rs4648006 | * | intron | G / A | 697 | 96 | 3 |  | 754 | 77 | 1 | 0.73 | 0.53 | 1.00 |  |
|  | rs13117745 |  | intron | G / A | 566 | 212 | 23 |  | 605 | 204 | 30 | 0.98 | 0.81 | 1.17 |  |
|  | rs4648022 |  | intron | G / A | 671 | 125 | 5 |  | 702 | 130 | 7 | 1.02 | 0.80 | 1.30 |  |
|  | rs4648037 | * | intron | A / G | 713 | 85 | 3 |  | 772 | 67 | 1 | 0.71 | 0.51 | 0.99 |  |
|  | rs11722146 |  | intron | G / A | 387 | 334 | 80 |  | 385 | 388 | 65 | 1.00 | 0.86 | 1.17 |  |
|  | rs4648090 |  | intron | G / A | 602 | 182 | 17 |  | 634 | 188 | 17 | 0.98 | 0.80 | 1.19 |  |
|  | rs4648110 |  | intron | T / A | 502 | 264 | 35 |  | 543 | 252 | 45 | 0.97 | 0.82 | 1.15 |  |
|  | rs230547 |  | intron | G / A | 651 | 142 | 8 |  | 681 | 152 | 6 | 0.99 | 0.78 | 1.25 |  |
|  | rs1609798 |  | intron | G / A | 357 | 357 | 87 |  | 363 | 396 | 80 | 1.00 | 0.86 | 1.16 |  |
|  | rs7674640 |  | flanking 3' UTR | A / G | 221 | 394 | 186 |  | 227 | 418 | 194 | 1.01 | 0.88 | 1.16 |  |
|  | rs997476 | * | flanking 3' UTR | C / A | 698 | 98 | 5 |  | 731 | 108 | 1 | 1.01 | 0.76 | 1.35 | **0.32** |
| **NFKB2 (chr 10: 104144219 - 104152271)** | | | |  |  |  |  |  |  |  |  |  |  |  |  |
|  | rs7897947 |  | intron | A / C | 501 | 258 | 40 |  | 536 | 268 | 34 | 0.94 | 0.79 | 1.11 |  |
|  | rs11574851 |  | coding (N698N) | G / A | 729 | 66 | 6 |  | 744 | 89 | 7 | 1.25 | 0.93 | 1.67 |  |
|  | rs1056890 |  | 3' UTR | G / A | 352 | 362 | 87 |  | 371 | 379 | 90 | 0.99 | 0.86 | 1.15 |  |
| **RELA (chr 11: 65178393 - 65186951)** | | | |  |  |  |  |  |  |  |  |  |  |  | **0.79** |
|  | rs10896027 |  | flanking 3' UTR | G / C | 320 | 372 | 108 |  | 348 | 375 | 117 | 0.98 | 0.85 | 1.13 |  |
|  | rs1049728 | * | flanking 3' UTR | G / C | 702 | 97 | 2 |  | 740 | 97 | 3 | 0.96 | 0.71 | 1.29 |  |
|  | rs11227247 |  | intron | A / C | 615 | 168 | 18 |  | 621 | 208 | 11 | 1.09 | 0.89 | 1.33 |  |
| **RELB (chr 19: 50196552 - 50233292)** | | | |  |  |  |  |  |  |  |  |  |  |  | **0.48** |
|  | rs35577563 |  | flanking 5' UTR | G / C | 320 | 365 | 116 |  | 312 | 407 | 121 | 1.06 | 0.92 | 1.22 |  |
|  | rs34647054 |  | intron | C / G | 405 | 327 | 68 |  | 424 | 345 | 70 | 1.00 | 0.86 | 1.16 |  |
|  | rs2288918 |  | intron | A / G | 321 | 363 | 117 |  | 299 | 418 | 123 | 1.10 | 0.96 | 1.27 |  |
|  | rs10856 | * | 3' UTR | C / A | 659 | 137 | 5 |  | 712 | 126 | 2 | 0.84 | 0.64 | 1.09 |  |
| **TBK1 (chr 12: 63132204 - 63182158)** | | | |  |  |  |  |  |  |  |  |  |  |  | **0.19** |
|  | rs10878175 |  | intron | G / C | 446 | 306 | 49 |  | 464 | 323 | 53 | 1.02 | 0.87 | 1.19 |  |
|  | rs7303577 |  | intron | A / C | 627 | 164 | 10 |  | 656 | 174 | 10 | 1.01 | 0.81 | 1.25 |  |
|  | rs7486100 |  | coding (I326I) | A / T | 249 | 389 | 163 |  | 250 | 426 | 164 | 1.01 | 0.88 | 1.16 |  |
|  | rs6581572 |  | flanking 3' UTR | G / A | 237 | 392 | 172 |  | 238 | 432 | 168 | 1.00 | 0.87 | 1.15 |  |
|  | rs11175419 |  | flanking 3' UTR | A / C | 633 | 161 | 7 |  | 695 | 139 | 6 | 0.80 | 0.63 | 1.01 |  |
| **TICAM1 (chr 19: 4766992 - 4769451)** | | | |  |  |  |  |  |  |  |  |  |  |  | **0.90** |
|  | rs1046673 |  | 3' UTR | G / A | 572 | 215 | 14 |  | 615 | 194 | 30 | 1.00 | 0.83 | 1.21 |  |
|  | rs2292151 |  | coding (D557D) | G / A | 461 | 287 | 53 |  | 472 | 310 | 58 | 1.04 | 0.89 | 1.22 |  |
|  | rs7255265 |  | coding (T4T) | G / A | 315 | 377 | 109 |  | 350 | 376 | 114 | 0.95 | 0.82 | 1.09 |  |
|  | rs10415014 |  | flanking 5' UTR | C / A | 590 | 191 | 20 |  | 612 | 213 | 15 | 1.00 | 0.82 | 1.22 |  |
| **TICAM2 (chr 5: 114942247 - 114989610)** | | | |  |  |  |  |  |  |  |  |  |  |  | **0.82** |
|  | rs2288384 |  | 3' UTR | G / A | 670 | 122 | 9 |  | 702 | 128 | 10 | 1.01 | 0.79 | 1.28 |  |
|  | rs10043094 |  | flanking 5' UTR | A / C | 305 | 393 | 103 |  | 336 | 390 | 114 | 0.97 | 0.84 | 1.12 |  |
|  | rs419939 |  | flanking 5' UTR | A / G | 418 | 317 | 66 |  | 430 | 353 | 57 | 0.99 | 0.85 | 1.15 |  |
|  | rs256946 |  | flanking 5' UTR | A / G | 621 | 166 | 14 |  | 655 | 169 | 16 | 0.98 | 0.80 | 1.21 |  |
|  | rs256962 |  | flanking 3' UTR | A / G | 207 | 401 | 193 |  | 218 | 421 | 200 | 0.99 | 0.86 | 1.14 |  |
|  | rs17474216 | * | 3' UTR | A / G | 722 | 76 | 3 |  | 769 | 69 | 2 | 0.84 | 0.60 | 1.18 |  |
|  | rs698365 |  | 3' UTR | C / A | 241 | 394 | 166 |  | 247 | 406 | 186 | 1.04 | 0.91 | 1.20 |  |
| **TIRAP (chr 11: 125658192 - 125670038)** | | | |  |  |  |  |  |  |  |  |  |  |  | **0.26** |
|  | rs646005 |  | intron | A / G | 305 | 380 | 116 |  | 302 | 402 | 135 | 1.08 | 0.94 | 1.24 |  |
|  | rs1893352 |  | intron | A / G | 593 | 191 | 17 |  | 591 | 223 | 26 | 1.20 | 0.99 | 1.45 |  |
|  | rs8177374 |  | coding (S180L) | G / A | 597 | 187 | 17 |  | 592 | 222 | 26 | 1.22 | 1.00 | 1.47 |  |
|  | rs8177376 |  | 3' UTR | A / C | 465 | 295 | 40 |  | 487 | 302 | 50 | 1.03 | 0.87 | 1.21 |  |
|  | rs625413 |  | 3' UTR | G / A | 481 | 276 | 44 |  | 507 | 291 | 42 | 0.98 | 0.83 | 1.15 |  |
|  | rs4937116 | * | flanking 3' UTR | G / A | 731 | 66 | 0 |  | 775 | 56 | 1 | 0.81 | 0.56 | 1.18 |  |
|  | rs1786704 |  | flanking 3' UTR | A / G | 459 | 312 | 30 |  | 534 | 262 | 44 | 0.87 | 0.74 | 1.03 |  |
|  | rs8177382 |  | flanking 3' UTR | C / A | 622 | 164 | 15 |  | 643 | 185 | 12 | 1.03 | 0.84 | 1.27 |  |
| **TLR3 (chr 4: 187227303 - 187243246)** | | | |  |  |  |  |  |  |  |  |  |  |  | **0.60** |
|  | rs5743305 |  | flanking 5' UTR | A / T | 308 | 382 | 111 |  | 333 | 396 | 111 | 0.96 | 0.83 | 1.11 |  |
|  | rs11721827 |  | intron | A / C | 586 | 195 | 20 |  | 605 | 210 | 25 | 1.06 | 0.88 | 1.28 |  |
|  | rs7657186 |  | intron | G / A | 473 | 285 | 43 |  | 525 | 278 | 37 | 0.88 | 0.75 | 1.04 |  |
|  | rs13126816 |  | intron | G / A | 452 | 307 | 42 |  | 477 | 322 | 39 | 0.97 | 0.82 | 1.14 |  |
|  | rs3775296 |  | UTR | C / A | 516 | 263 | 22 |  | 544 | 261 | 34 | 1.03 | 0.86 | 1.23 |  |
|  | rs7668666 |  | intron | C / A | 424 | 330 | 47 |  | 445 | 334 | 58 | 1.02 | 0.87 | 1.20 |  |
|  | rs3775292 |  | intron | G / C | 509 | 262 | 29 |  | 522 | 284 | 34 | 1.06 | 0.89 | 1.26 |  |
|  | rs3775291 |  | coding (L412F) | G / A | 418 | 318 | 65 |  | 427 | 348 | 65 | 1.02 | 0.88 | 1.19 |  |
|  | rs10025405 |  | flanking 3' UTR | A / G | 222 | 376 | 145 |  | 240 | 372 | 157 | 0.99 | 0.86 | 1.14 |  |
|  | rs4862633 |  | flanking 3' UTR | G / A | 455 | 289 | 57 |  | 481 | 312 | 47 | 0.95 | 0.81 | 1.11 |  |
|  | rs4608848 |  | flanking 3' UTR | A / G | 295 | 373 | 133 |  | 314 | 401 | 125 | 0.95 | 0.83 | 1.10 |  |
| **TLR4 (chr 9: 119506431 - 119519589)** | | | |  |  |  |  |  |  |  |  |  |  |  | **0.75** |
|  | rs2770150 |  | flanking 5' UTR | A / G | 423 | 317 | 61 |  | 437 | 348 | 55 | 0.99 | 0.85 | 1.16 |  |
|  | rs1927914 |  | flanking 5' UTR | A / G | 343 | 383 | 75 |  | 356 | 392 | 92 | 1.05 | 0.91 | 1.22 |  |
|  | rs11536869 | * | intron | A / G | 747 | 52 | 2 |  | 769 | 71 | 0 | 1.29 | 0.89 | 1.86 |  |
|  | rs1927911 |  | intron | G / A | 432 | 327 | 40 |  | 437 | 341 | 61 | 1.12 | 0.96 | 1.32 |  |
|  | rs12377632 |  | intron | T / C | 274 | 355 | 112 |  | 295 | 359 | 108 | 0.94 | 0.81 | 1.09 |  |
|  | rs1927907 |  | intron | G / A | 554 | 180 | 11 |  | 561 | 189 | 19 | 1.11 | 0.90 | 1.36 |  |
|  | rs4986791 | * | coding (T399I) | G / A | 706 | 91 | 3 |  | 750 | 87 | 3 | 0.90 | 0.66 | 1.22 |  |
|  | rs11536889 |  | 3' UTR | G / C | 584 | 197 | 20 |  | 612 | 204 | 24 | 1.02 | 0.84 | 1.23 |  |
|  | rs11536897 | * | flanking 3' UTR | G / A | 723 | 77 | 1 |  | 753 | 84 | 2 | 1.06 | 0.77 | 1.47 |  |
|  | rs1927906 |  | flanking 3' UTR | A / G | 658 | 136 | 7 |  | 685 | 146 | 9 | 1.04 | 0.83 | 1.32 |  |
|  | rs1554973 |  | flanking 3' UTR | A / G | 447 | 313 | 41 |  | 457 | 336 | 46 | 1.05 | 0.89 | 1.23 |  |
|  | rs7044464 |  | flanking 3' UTR | T / A | 576 | 205 | 20 |  | 592 | 231 | 17 | 1.04 | 0.86 | 1.26 |  |
|  | rs7037225 |  | flanking 3' UTR | C / T | 529 | 202 | 13 |  | 533 | 217 | 20 | 1.11 | 0.91 | 1.35 |  |
| **TLR7 (chr X: 12795123 - 12818401)** | | | |  |  |  |  |  |  |  |  |  |  |  | **0.15** |
|  | rs2302267 | * | intron | A / C | 718 | 79 | 4 |  | 731 | 103 | 6 | 1.29 | 0.95 | 1.74 |  |
|  | rs5741880 |  | intron | C / A | 648 | 145 | 8 |  | 694 | 141 | 5 | 0.88 | 0.70 | 1.12 |  |
|  | rs179019 |  | intron | C / A | 479 | 282 | 39 |  | 459 | 324 | 56 | 1.21 | 1.03 | 1.43 |  |
|  | rs5743749 | * | intron | G / A | 688 | 106 | 7 |  | 736 | 100 | 4 | 0.86 | 0.65 | 1.15 |  |
|  | rs1634320 |  | intron | G / A | 646 | 144 | 11 |  | 700 | 135 | 5 | 0.82 | 0.65 | 1.03 |  |
|  | rs179012 |  | intron | G / A | 401 | 353 | 46 |  | 420 | 352 | 68 | 1.07 | 0.91 | 1.25 |  |
|  | rs5743774 |  | intron | C / A | 798 | 3 | 0 |  | 834 | 6 | 0 | 1.39 | 0.69 | 2.79 |  |
|  | rs179010 |  | intron | G / A | 383 | 355 | 63 |  | 377 | 375 | 86 | 1.13 | 0.98 | 1.32 |  |
|  | rs179008 |  | coding (Q11L) | T / A | 488 | 286 | 27 |  | 506 | 296 | 37 | 1.05 | 0.89 | 1.25 |  |
|  | rs864058 |  | coding (T801T) | G / A | 678 | 118 | 5 |  | 707 | 124 | 9 | 1.06 | 0.83 | 1.36 |  |
|  | rs3853839 |  | 3' UTR | C / G | 501 | 224 | 20 |  | 518 | 220 | 20 | 0.96 | 0.79 | 1.16 |  |
| **TNF (chr 6: 31651329 - 31654091)** | | | |  |  |  |  |  |  |  |  |  |  |  | **0.88** |
|  | rs2009658 |  | flanking 5' UTR | C / G | 574 | 207 | 20 |  | 608 | 210 | 22 | 0.98 | 0.81 | 1.18 |  |
|  | rs915654 |  | flanking 5' UTR | T / A | 346 | 353 | 102 |  | 371 | 357 | 112 | 0.99 | 0.86 | 1.14 |  |
|  | rs2239704 |  | 5' UTR | C / A | 310 | 365 | 126 |  | 329 | 396 | 115 | 0.95 | 0.83 | 1.09 |  |
|  | rs2229094 |  | coding (C13R) | A / G | 451 | 294 | 56 |  | 465 | 317 | 58 | 1.02 | 0.87 | 1.19 |  |
|  | rs2229092 | * | coding (H51P) | A / C | 667 | 71 | 1 |  | 681 | 81 | 5 | 1.17 | 0.84 | 1.63 |  |
|  | rs1799964 |  | flanking 3' UTR | A / G | 517 | 246 | 37 |  | 533 | 270 | 36 | 1.02 | 0.86 | 1.21 |  |
|  | rs1800610 |  | intron | G / A | 676 | 115 | 10 |  | 698 | 135 | 7 | 1.06 | 0.83 | 1.34 |  |
|  | rs3093662 |  | intron | A / G | 702 | 93 | 5 |  | 720 | 114 | 6 | 1.17 | 0.89 | 1.53 |  |
|  | rs769178 |  | flanking 3' UTR | C / A | 678 | 113 | 10 |  | 700 | 134 | 6 | 1.05 | 0.83 | 1.34 |  |
|  | rs2256965 |  | intron | G / A | 279 | 387 | 134 |  | 298 | 405 | 137 | 0.98 | 0.85 | 1.13 |  |
|  | rs1052248 |  | 3' UTR | T / A | 443 | 302 | 56 |  | 456 | 328 | 55 | 1.01 | 0.87 | 1.18 |  |
|  | rs11575839 | * | coding (S52S) | C / T | 700 | 36 | 2 |  | 731 | 35 | 0 | 0.88 | 0.55 | 1.41 |  |
| **TNFRSF1A (chr 12: 6308184 - 6321522)** | | | |  |  |  |  |  |  |  |  |  |  |  | **0.87** |
|  | rs1800693 |  | intron | A / G | 285 | 391 | 124 |  | 307 | 388 | 143 | 1.01 | 0.88 | 1.16 |  |
|  | rs4149584 |  | coding (R121P) | G / A | 773 | 28 | 0 |  | 804 | 36 | 0 | 1.11 | 0.86 | 1.43 |  |
|  | rs4149575 |  | intron | C / A | 672 | 122 | 7 |  | 711 | 124 | 5 | 0.94 | 0.73 | 1.20 |  |
|  | rs767455 |  | coding (P12P) | A / G | 259 | 399 | 142 |  | 285 | 406 | 149 | 0.97 | 0.84 | 1.11 |  |
|  | rs4149570 |  | flanking 5' UTR | C / A | 282 | 396 | 123 |  | 302 | 389 | 149 | 1.03 | 0.90 | 1.19 |  |
| **TNFRSF1B (chr 1: 12149647 - 12191864)** | | | |  |  |  |  |  |  |  |  |  |  |  | **0.37** |
|  | rs652625 | * | flanking 5' UTR | T / A | 707 | 88 | 6 |  | 754 | 83 | 3 | 0.86 | 0.63 | 1.17 |  |
|  | rs1148458 |  | intron | G / A | 693 | 102 | 6 |  | 709 | 126 | 5 | 1.15 | 0.89 | 1.49 |  |
|  | rs976881 |  | intron | G / A | 369 | 344 | 88 |  | 397 | 365 | 77 | 0.93 | 0.80 | 1.08 |  |
|  | rs499646 | * | intron | G / A | 720 | 76 | 5 |  | 766 | 71 | 2 | 0.85 | 0.61 | 1.18 |  |
|  | rs5745981 | * | intron | A / T | 764 | 37 | 0 |  | 797 | 40 | 3 | 1.11 | 0.71 | 1.75 |  |
|  | rs816050 |  | intron | G / A | 520 | 244 | 37 |  | 576 | 235 | 28 | 0.85 | 0.71 | 1.01 |  |
|  | rs587406 |  | intron | A / C | 486 | 272 | 43 |  | 485 | 299 | 54 | 1.11 | 0.95 | 1.30 |  |
|  | rs17882988 |  | intron | G / A | 595 | 186 | 20 |  | 586 | 223 | 30 | 1.22 | 1.01 | 1.47 |  |
|  | rs5746012 | * | intron | C / A | 750 | 50 | 1 |  | 797 | 40 | 1 | 0.76 | 0.49 | 1.15 |  |
|  | rs5746014 |  | intron | A / G | 649 | 141 | 11 |  | 672 | 158 | 10 | 1.05 | 0.84 | 1.31 |  |
|  | rs1061622 |  | coding (M196R) | A / C | 490 | 274 | 37 |  | 502 | 289 | 49 | 1.08 | 0.91 | 1.27 |  |
|  | rs5746026 | * | coding (E232K) | G / A | 755 | 51 | 1 |  | 801 | 43 | 1 | 0.80 | 0.53 | 1.20 |  |
|  | rs2275416 |  | intron | G / A | 531 | 245 | 25 |  | 535 | 263 | 41 | 1.14 | 0.96 | 1.36 |  |
|  | rs5746051 |  | intron | A / G | 559 | 222 | 20 |  | 566 | 241 | 33 | 1.14 | 0.95 | 1.37 |  |
|  | rs1061624 |  | 3' UTR | G / A | 209 | 351 | 195 |  | 239 | 357 | 204 | 0.95 | 0.83 | 1.09 |  |
|  | rs3397 |  | 3' UTR | T / C | 286 | 342 | 108 |  | 302 | 356 | 107 | 0.97 | 0.84 | 1.12 |  |
|  | rs5746065 |  | 3' UTR | C / A | 657 | 136 | 8 |  | 720 | 108 | 12 | 0.82 | 0.65 | 1.04 |  |
|  | rs1061628 |  | 3' UTR | G / A | 304 | 377 | 120 |  | 308 | 398 | 133 | 1.04 | 0.91 | 1.20 |  |
|  | rs1061631 |  | 3' UTR | G / A | 467 | 239 | 31 |  | 480 | 238 | 40 | 1.03 | 0.87 | 1.23 |  |
| **TOLLIP (chr 11: 1252177 - 1287415)** | | | |  |  |  |  |  |  |  |  |  |  |  | **0.13** |
|  | rs5744038 | * | flanking 3' UTR | A / G | 693 | 107 | 1 |  | 736 | 95 | 9 | 0.90 | 0.68 | 1.21 |  |
|  | rs5744037 | * | flanking 3' UTR | G / A | 716 | 84 | 1 |  | 749 | 88 | 3 | 1.02 | 0.75 | 1.40 |  |
|  | rs5744036 | * | 3' UTR | G / A | 746 | 55 | 0 |  | 780 | 57 | 3 | 1.04 | 0.71 | 1.52 |  |
|  | rs5744034 |  | 3' UTR | A / G | 544 | 227 | 30 |  | 565 | 253 | 22 | 0.98 | 0.82 | 1.18 |  |
|  | rs3168046 |  | 3' UTR | G / A | 245 | 397 | 159 |  | 260 | 402 | 178 | 1.02 | 0.89 | 1.17 |  |
|  | rs5744030 | * | 3' UTR | G / A | 747 | 54 | 0 |  | 787 | 50 | 3 | 0.93 | 0.63 | 1.38 |  |
|  | rs5744013 | * | intron | A / G | 716 | 84 | 1 |  | 749 | 88 | 3 | 1.02 | 0.75 | 1.40 |  |
|  | rs5743961 | * | intron | G / A | 717 | 82 | 0 |  | 748 | 89 | 1 | 1.05 | 0.77 | 1.44 |  |
|  | rs5743937 |  | intron | A / G | 561 | 224 | 16 |  | 575 | 237 | 28 | 1.11 | 0.92 | 1.33 |  |
|  | rs5743925 |  | intron | A / C | 792 | 9 | 0 |  | 824 | 16 | 0 | 1.70 | 0.75 | 3.86 |  |
|  | rs4963060 |  | intron | G / A | 379 | 348 | 74 |  | 392 | 358 | 90 | 1.05 | 0.91 | 1.22 |  |
|  | rs5743915 |  | intron | G / A | 260 | 396 | 144 |  | 310 | 402 | 128 | 0.86 | 0.75 | 0.99 |  |
|  | rs5743899 |  | intron | A / G | 515 | 261 | 25 |  | 526 | 274 | 40 | 1.11 | 0.93 | 1.31 |  |
|  | rs5743890 |  | intron | A / G | 590 | 198 | 13 |  | 608 | 210 | 22 | 1.10 | 0.90 | 1.33 |  |
|  | rs5743885 |  | intron | A / G | 691 | 104 | 6 |  | 699 | 135 | 6 | 1.22 | 0.95 | 1.58 |  |
|  | rs5743880 | * | intron | G / A | 782 | 19 | 0 |  | 817 | 22 | 1 | 1.16 | 0.63 | 2.15 |  |
|  | rs5743859 |  | intron | G / A | 499 | 266 | 35 |  | 567 | 251 | 22 | 0.80 | 0.67 | 0.95 |  |
|  | rs5743856 | * | intron | A / G | 750 | 51 | 0 |  | 794 | 44 | 2 | 0.85 | 0.56 | 1.28 |  |
|  | rs5743854 |  | flanking 5' UTR | C / G | 642 | 151 | 8 |  | 658 | 169 | 13 | 1.13 | 0.90 | 1.40 |  |
|  | rs5743851 | * | flanking 5' UTR | A / G | 658 | 139 | 4 |  | 708 | 130 | 1 | 0.85 | 0.66 | 1.10 |  |
| **TRAF3 (chr 14: 102313569 - 102442381)** | | | |  |  |  |  |  |  |  |  |  |  |  | **0.98** |
|  | rs10137035 |  | intron | G / A | 478 | 287 | 36 |  | 498 | 297 | 45 | 1.04 | 0.88 | 1.22 |  |
|  | rs941726 |  | intron | G / A | 576 | 207 | 18 |  | 596 | 222 | 22 | 1.05 | 0.87 | 1.27 |  |
|  | rs12436181 |  | intron | G / A | 322 | 380 | 99 |  | 348 | 388 | 104 | 0.97 | 0.84 | 1.13 |  |
|  | rs12147254 |  | intron | G / A | 407 | 325 | 69 |  | 404 | 370 | 66 | 1.05 | 0.90 | 1.22 |  |
|  | rs1956160 |  | intron | G / A | 244 | 406 | 151 |  | 251 | 414 | 175 | 1.05 | 0.92 | 1.21 |  |
|  | rs2144825 |  | intron | C / A | 509 | 267 | 25 |  | 539 | 273 | 28 | 0.99 | 0.83 | 1.18 |  |
|  | rs7145509 |  | intron | C / A | 455 | 309 | 37 |  | 498 | 291 | 49 | 0.96 | 0.82 | 1.13 |  |
|  | rs12898022 |  | intron | G / A | 338 | 378 | 84 |  | 366 | 377 | 94 | 0.98 | 0.85 | 1.14 |  |
|  | rs12588538 |  | intron | A / G | 528 | 251 | 22 |  | 570 | 239 | 31 | 0.97 | 0.81 | 1.15 |  |
|  | rs4906269 |  | intron | A / C | 526 | 253 | 22 |  | 562 | 247 | 31 | 0.99 | 0.83 | 1.18 |  |
|  | rs10132870 | * | intron | A / C | 764 | 37 | 0 |  | 803 | 36 | 1 | 0.95 | 0.59 | 1.51 |  |
|  | rs4906271 | * | flanking 3' UTR | A / T | 716 | 81 | 4 |  | 747 | 89 | 3 | 1.04 | 0.76 | 1.42 |  |
| **TRAF6 (chr 11: 36467302 - 36488398)** | | | |  |  |  |  |  |  |  |  |  |  |  | **0.39** |
|  | rs5030485 |  | flanking 3' UTR | C / A | 604 | 182 | 15 |  | 624 | 202 | 14 | 1.04 | 0.85 | 1.27 |  |
|  | rs5030472 |  | intron | G / A | 626 | 165 | 10 |  | 658 | 171 | 10 | 0.98 | 0.79 | 1.22 |  |
|  | rs5030437 |  | intron | G / A | 570 | 209 | 22 |  | 590 | 228 | 22 | 1.03 | 0.85 | 1.24 |  |
|  | rs5030419 |  | intron | G / C | 622 | 167 | 11 |  | 623 | 206 | 11 | 1.18 | 0.95 | 1.45 |  |
|  | rs5030411 |  | flanking 5' UTR | A / G | 310 | 370 | 121 |  | 300 | 420 | 120 | 1.05 | 0.91 | 1.21 |  |
|  | rs5030409 |  | flanking 5' UTR | G / A | 610 | 174 | 17 |  | 625 | 200 | 15 | 1.06 | 0.87 | 1.30 |  |
| **UBE2C (chr 20: 43874662 - 43879003)** | | | |  |  |  |  |  |  |  |  |  |  |  | **0.45** |
|  | rs399672 |  | intron | A / G | 419 | 318 | 64 |  | 465 | 311 | 64 | 0.92 | 0.79 | 1.07 |  |
|  | rs12625621 |  | flanking 5' UTR | G / A | 305 | 369 | 127 |  | 321 | 388 | 131 | 0.99 | 0.86 | 1.14 |  |
|  | rs3848711 |  | flanking 3' UTR | A / G | 281 | 378 | 142 |  | 288 | 405 | 146 | 1.01 | 0.88 | 1.16 |  |
| **UBE3A (chr 15: 23133489 - 23235221)** | | | |  |  |  |  |  |  |  |  |  |  |  | **0.50** |
|  | rs8179187 | * | intron | A / C | 681 | 116 | 4 |  | 726 | 107 | 7 | 0.89 | 0.68 | 1.18 |  |
|  | rs1041933 |  | intron | A / G | 497 | 264 | 40 |  | 519 | 277 | 44 | 1.02 | 0.86 | 1.20 |  |
|  | rs17115585 |  | intron | A / T | 626 | 161 | 14 |  | 636 | 187 | 17 | 1.13 | 0.92 | 1.39 |  |
| **VISA (chr 20: 3775484 - 3795973)** | | | |  |  |  |  |  |  |  |  |  |  |  | **0.11** |
|  | rs4815617 |  | flanking 5' UTR | G / A | 645 | 148 | 8 |  | 672 | 160 | 8 | 1.03 | 0.82 | 1.29 |  |
|  | rs6116065 |  | intron | G / A | 371 | 342 | 88 |  | 359 | 383 | 98 | 1.10 | 0.95 | 1.27 |  |
|  | rs6084497 |  | intron | A / G | 425 | 321 | 54 |  | 450 | 321 | 67 | 1.02 | 0.87 | 1.18 |  |
|  | rs8116776 |  | intron | G / A | 462 | 307 | 32 |  | 481 | 304 | 55 | 1.08 | 0.92 | 1.28 |  |
|  | rs6133067 |  | intron | G / A | 436 | 302 | 61 |  | 484 | 304 | 52 | 0.89 | 0.76 | 1.04 |  |
|  | rs7262903 |  | coding (Q198K) | C / A | 590 | 192 | 18 |  | 590 | 238 | 11 | 1.11 | 0.91 | 1.35 |  |
|  | rs914294 |  | intron | G / A | 590 | 193 | 18 |  | 657 | 172 | 11 | 0.78 | 0.64 | 0.96 |  |
| **ZBP1 (chr 20: 55612308 - 55628935)** | | | |  |  |  |  |  |  |  |  |  |  |  | **0.82** |
|  | rs761344 |  | flanking 3' UTR | A / T | 231 | 418 | 152 |  | 249 | 425 | 166 | 1.00 | 0.87 | 1.15 |  |
|  | rs6064572 |  | 3' UTR | C / A | 313 | 375 | 113 |  | 352 | 357 | 131 | 0.97 | 0.85 | 1.12 |  |
|  | rs6025653 |  | intron | A / C | 309 | 372 | 120 |  | 331 | 389 | 120 | 0.97 | 0.84 | 1.11 |  |
|  | rs6070180 |  | intron | G / A | 340 | 352 | 109 |  | 371 | 364 | 105 | 0.94 | 0.82 | 1.08 |  |
|  | rs742724 |  | intron | G / A | 661 | 134 | 6 |  | 680 | 143 | 15 | 1.14 | 0.91 | 1.44 |  |
|  | rs2073145 |  | coding (E88K) | A / G | 380 | 340 | 81 |  | 380 | 374 | 85 | 1.05 | 0.91 | 1.22 |  |
|  | rs4811890 |  | intron | G / A | 575 | 204 | 22 |  | 595 | 226 | 19 | 1.02 | 0.84 | 1.23 |  |

*^a^ All models are adjusted for continuous linear age at reference.*

*^*^ Dominant model, all others are log-additive.*
